# Microarray analysis of retinal gene expression in *Egr-1* knockout mice

**Published:** 2009-12-10

**Authors:** Ruth Schippert, Frank Schaeffel, Marita Pauline Feldkaemper

**Affiliations:** Institute for Ophthalmic Research, Section of Neurobiology of the Eye, University Eye Hospital Tuebingen, Tuebingen, Germany

## Abstract

**Purpose:**

We found earlier that 42 day-old *Egr-1* knockout mice had longer eyes and a more myopic refractive error compared to their wild-types. To identify genes that could be responsible for the temporarily enhanced axial eye growth, a microarray analysis was performed in knockout and wild-type mice at the postnatal ages of 30 and 42 days.

**Methods:**

The retinas of homozygous and wild-type *Egr-1* knockout mice (Taconic, Ry, Denmark) were prepared for RNA isolation (RNeasy Mini Kit, Qiagen) at the age of 30 or 42 days, respectively (n=12 each). Three retinas were pooled and labeled cRNA was made. The samples were hybridized to Affymetrix GeneChip Mouse Genome 430 2.0 Arrays. Hybridization signals were calculated using GC-RMA normalization. Genes were identified as differentially expressed if they showed a fold-change (FC) of at least 1.5 and a p-value <0.05. A false-discovery rate of 5% was applied. Ten genes with potential biologic relevance were examined further with semiquantitative real-time RT–PCR.

**Results:**

Comparing mRNA expression levels between wild-type and homozygous *Egr-1* knockout mice, we found 73 differentially expressed genes at the age of 30 days and 135 genes at the age of 42 days. Testing for differences in gene expression between the two ages (30 versus 42 days), 54 genes were differently expressed in wild-type mice and 215 genes in homozygous animals. Based on three networks proposed by Ingenuity pathway analysis software, nine differently expressed genes in the homozygous *Egr-1* knockout mice were chosen for further validation by real-time RT–PCR, three genes in each network. In addition, the gene that was most prominently regulated in the knockout mice, compared to wild-type, at both 30 days and 42 days of age (protocadherin beta-9 [*Pcdhb9*]), was tested with real-time RT–PCR. Changes in four of the ten genes could be confirmed by real-time RT–PCR: nuclear prelamin A recognition factor (*Narf*), oxoglutarate dehydrogenase (*Ogdh*), selenium binding protein 1 (*Selenbp1*), and *Pcdhb9*. Except for *Pcdhb9*, the genes whose mRNA expression levels were validated were listed in one of the networks proposed by Ingenuity pathway analysis software. In addition to these genes, the software proposed several key-regulators which did not change in our study: retinoic acid, vascular endothelial growth factor A (*VEGF-A*), FBJ murine osteosarcoma viral oncogene homolog (*cFos*), and others.

**Conclusions:**

Identification of genes that are differentially regulated during the development period between postnatal day 30 (when both homozygous and wild-type mice still have the same axial length) and day 42 (where the difference in eye length is apparent) could improve the understanding of mechanisms for the control of axial eye growth and may lead to potential targets for pharmacological intervention. With the aid of pathway-analysis software, a coarse picture of possible biochemical pathways could be generated. Although the mRNA expression levels of proteins proposed by the software, like *VEGF*, *FOS*, retinoic acid (RA) receptors, or cellular RA binding protein, did not show any changes in our experiment, these molecules have previously been implicated in the signaling cascades controlling axial eye growth. According to the pathway-analysis software, they represent links between several proteins whose mRNA expression was changed in our study.

## Introduction

Myopia is becoming an increasing problem, especially in industrial nations. It is widely believed that both hereditary and environmental factors contribute to the development of myopia. Several molecules have already been identified in the retina that appear to be involved in the visual control of axial elongation of the eye (e.g., dopamine [1-3], retinoic acid [4-6], nitric oxide [7-9], vasoactive intestinal polypeptide [10-12]). Another factor that was found to be involved was the transcription factor Egr-1 (early growth response protein-1), the mammalian ortholog to the avian protein ZENK (also called Tis8, Ngfi-A, Kro×−24, Zif268 in other species). By means of immunocytochemistry it was initially found that the number of ZENK-immunoreactive amacrine cells in the retina of chicks is increased under conditions that lead to a reduction in eye growth (myopic defocus, recovery of myopia) and decreased under conditions that enhance ocular growth (hyperopic defocus, form-deprivation). These changes were most prominent and distinct in a specific subset of amacrine cells, the glucagon-containing amacrine cells [13,14]. Recently, this bi-directional response was detected by means of immunohistochemistry in another glucagon-containing cell type of the chicken retina as well, the so-called bullwhip-cells [15]. Moreover, a downregulation of *Egr-1* mRNA in total retinal tissue was found in mice after short periods of form-deprivation [16]. All of these experiments suggested that Egr-1 (ZENK) is an important factor in controlling eye growth, at least in some animal models for myopia. However, it should be noticed that mRNA levels of *Egr-1* in the total retina of chicks do not seem to show this bidirectional response consistently. Although *ZENK* mRNA levels are upregulated in total retinal samples within one hour after diffuser removal of former form-deprived chicks (recovery of myopia) [17], treatment with both minus lenses and plus lenses for one day reduced the amount of *ZENK* mRNA in the total retina of chicks in both cases, suggesting that the role of *Egr-1* is complex and may vary among special cell types [18,19]. Unfortunately, no study is available that investigated the time course of *Egr-1* mRNA changes in detail. Therefore, current knowledge about the regulation of *Egr-1* during increased or decreased eye growth is still limited.

Studies on *Egr-1* knockout mice were in line with the hypothesis that *Egr-1* has a function in the regulation of eye growth. Homozygous knockout mice, lacking functional Egr-1 protein, developed relative axial myopia at the age of 42 and 56 days (compared to heterozygous and wild-type *Egr-1* knockout mice [20]). The difference in axial length declined with increasing age, but the differences in the refractive state persisted. Paraxial schematic eye modeling suggested that other optical elements, possibly the lens, had also changed in the *Egr-1* knockout mice. This is not surprising, given that Egr-1 was absent not only from retinal amacrine cells but from all cells of the body. The effect of lacking Egr-1 protein should have long-ranging effects on other cells in the retina, eye, and the autonomic nervous system or the endocrine system.

Egr-1 is known to have a function in a variety of biologic processes (e.g., cell proliferation [21], brain plasticity and learning [22], apoptosis [23]) and several target genes of Egr-1 have already been identified. *Egr-1*-overexpression in synovial fibroblasts leads to an increased expression of collagen type 1 and of tissue inhibitor of metalloproteinases type 1 and 3 (TIMP1 and TIMP3) [24]. Since the induction of myopia is associated with scleral thinning through reduced accumulation of collagen and increased degradation of scleral tissue [25-27], the reduction of Egr-1-stimulated collagen expression and the reduced inhibition of degrading enzymes (such as the matrix-metalloproteinases that are repressed by TIMPs) that could take place in animals without functional Egr-1 protein, could explain the myopic phenotype of these mice. Other genes that are already known to be influenced by Egr-1 are for instance platelet-derived growth factor-A and -B (*PDGF-A* and *-B*) [28,29], basic fibroblast growth factor (*bFGF*) [30] and transforming growth factor-beta (*TGF-β*) [31].

Because of the complex role of *Egr-1* in the regulation of various other proteins, and the differences in axial eye length between the Egr-1 knockout mice and the wild-type mice, we have studied the role of Egr-1 in the retina in more detail. Retinal samples of *Egr-1* knockout and wild-type mice at the age of 30 days (no difference in axial eye length yet) and 42 days (already a difference in axial eye length of 59 µm) were compared regarding their mRNA expression changes, both between the two genotypes and within the same genotype between the two age groups.

## Methods

### Animals

Experiments were conducted in accordance with the ARVO Statement for the Use of Animals in Ophthalmic and Vision Research and were approved by the University Commission for Animal Welfare. Egr-1 knockout mice, generated on C57/BL6 background, were purchased from Taconic (Ry, Denmark) and bred in the animal facilities of the institute after a breeding permission was obtained from the company. Since female homozygous knockout mice are sterile because of a deficiency of luteinizing hormone-beta (which is due to the lack of Egr-1 [32]), only heterozygous females were bred. Animals were housed in standard cages with their littermates under a 12 h light/dark cycle with unrestricted access to water and food pellets. Illumination was provided by standard fluorescent lamps and was approximately 200 lx. Standard PCR was performed to determine genotype (specific primer sequences provided by Taconic) and gender (with primers designed for the gene encoding the sex-determining region Y represents as SRY).

Male mice were killed by an overdose of diethyl ether at the mean age of 30 days (p29-p31) or 42 days (p41-p43). Eyes were enucleated and retinas were prepared carefully to ensure that the samples were not contaminated with retinal pigment epithelium. Tissue was snap-frozen in liquid nitrogen. The retinas of 12 homozygous Egr-1 knockout mice (hm) and 12 wild-type mice (wt) of the same strain were prepared for both time points (48 animals in total). Three single retinas from different mice were then pooled to obtain four samples per group (wt/30, wt/42, hm/30, hm/42), and RNA was isolated using the RNeasy Mini Kit (Qiagen, Hilden, Germany) according to the manufacturer's instructions.

### Microarray

Quality check of RNA, cDNA synthesis and labeling and the actual microarray analysis was performed by the Affymetrix Resource Facility at the University of Tuebingen. The quality of total RNA was monitored by Agilent 2100 Bioanalyzer (Agilent Technologies, Palo Alto, CA) following the manufacturer's instructions. Generation of double-stranded cDNA, preparation and labeling of cRNA, hybridization to 430 2.0 Mouse Genome Arrays (Affymetrix, Santa Clara, CA), washing, and scanning were performed according to the standard Affymetrix protocol. Scanning and analysis were performed using the Affymetrix Microarray Suite Software (version 5.0) and the signal intensities were analyzed using ArrayAssist 5.5.1 (Stratagene, La Jolla, CA).

Data were normalized using the GC-RMA normalization method which uses the GC content of the probes in normalization with RMA (Robust Multi-Array). To correct for multiple testing, a false-discovery rate of 5% was applied. All comparisons of mRNA expression levels between the groups were performed using un-paired *t*-tests. Genes were identified as differentially expressed if they showed a fold-change (FC) of at least 1.5 with a p value lower than 0.05. Fold change was calculated by dividing the experimental value (lens-treated, t) by the control value (untreated control, c). If the relative signal intensity of the control was higher than the intensity of the treated samples, the negative reciprocal was calculated (-c/t). A fold change of 1 or −1 therefore indicates no change, while a fold change of 2 equals a doubling in product, and a fold change of −2 equals a halving in transcript abundance.

The data discussed in this publication have been deposited in the National Center for Biotechnology Information (NCBI's) Gene Expression Omnibus (GEO) and are accessible through GEO Series accession number GSE16974.

### Pathway analysis

The list of differently expressed genes was subjected to a subsequent post-analysis task to find the main biologic processes associated with the experimental system. The “Ingenuity Pathways Analysis” Software 5.0 (IPA, Ingenuity Systems) was applied to elucidate putative pathways associated with the gene expression changes in the retinas of the *Egr-1* knockout mice between the age of 30 days and 42 days. For this purpose, 215 genes which were classed as “differently expressed,” e.g., those whose retinal mRNA expression in the knockout mice at p30 was significantly different from the expression at p42, were analyzed and theoretical networks and pathways were computed. The IPA is a manually curated database of functional interactions and contains previously published findings from peer-reviewed publications. Interactions between proteins and molecules in the proposed networks are therefore supported by published information which is associated by the program with known biologic pathways. It should be noted here that the interactions presented in the networks are not specific for the retina or brain tissue, as the database contains literature from many different research areas. If the mRNA expression levels of many proteins present in one proposed network have actually been found to be changed, it is likely that they are connected with each other and that their changes may represent a response to the lack of Egr-1.

### Real-time RT–PCR

**Figure 1:**
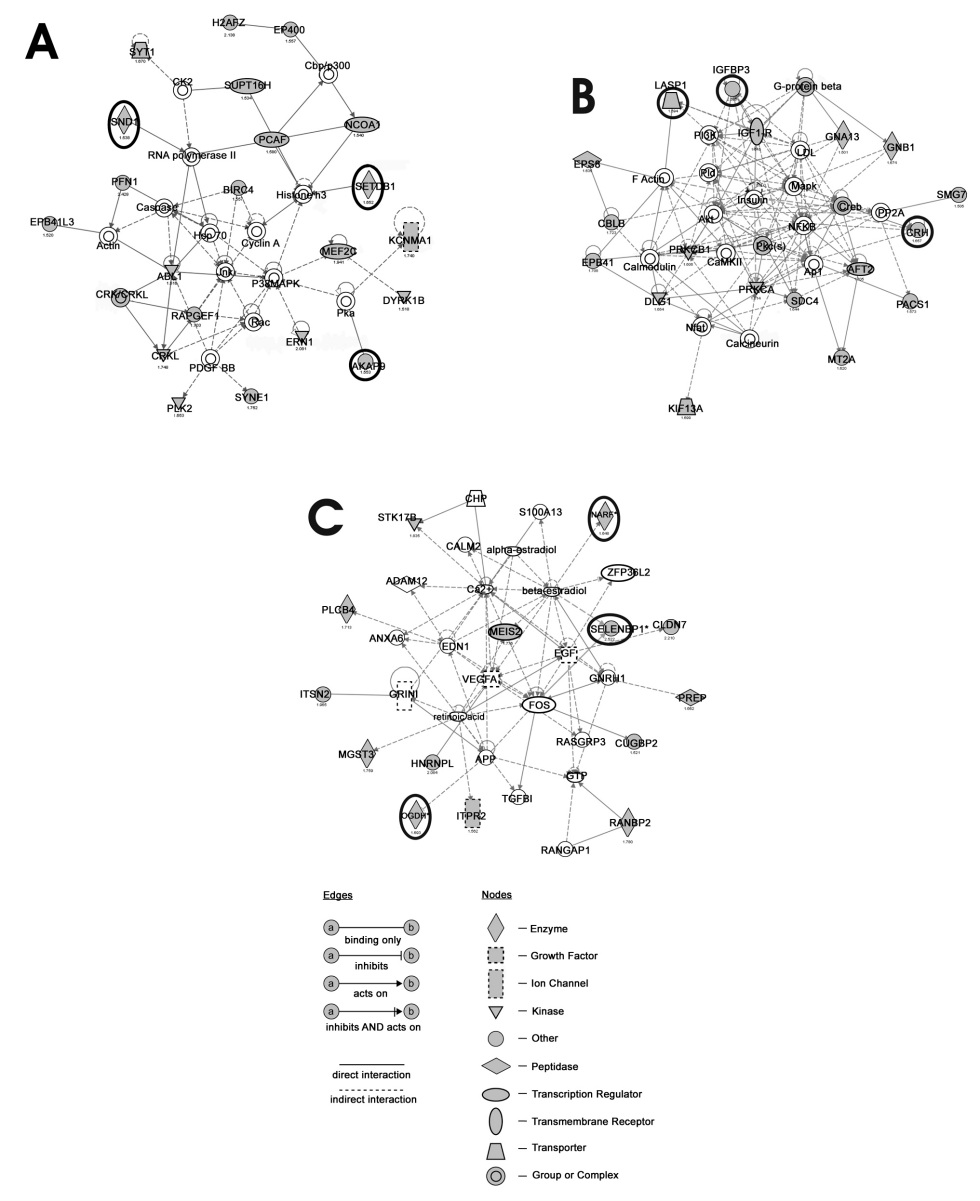
Networks predicted by Ingenuity Pathway Analysis in the homozygous Egr-1 knockout mice. Networks proposed by Ingenuity Pathways Analysis Software. All genes whose mRNA expression levels were found to be differentially regulated in the knockout mice between the age of 30 days and 42 days are highlighted in gray. Encircled are those genes that were chosen for validation by real-time RT–PCR. A detailed legend describing the symbols used in this scheme is enclosed in the figure. Asterisks denote changes in gene expression that were validated using real-time PCR.

Based on three networks found in the homozygous *Egr-1* knockout mice computed by Ingenuity Pathways Analysis Software (hm/30 versus hm/42, see Figure 1A-C), nine genes were chosen for further validation of their expression changes by real-time RT–PCR (three genes per network). For network A we chose: A kinase anchor protein 9 (*Akap9*), SET domain, bifurcated 1 (*Setdb1*) and staphylococcal nuclease domain containing 1 (*Snd1*). The three genes representing network B were: corticotropin releasing hormone (*Crh*), insulin-like growth factor binding protein 3 (*Igfbp3*), and LIM and SH3 protein 1 (*Lasp1*). Finally, from network C we chose: nuclear prelamin A recognition factor (*Narf*), oxoglutarate dehydrogenase (*Ogdh*), and selenium binding protein 1 (*Selenbp1*). In addition, protocadherin-beta 9 (*Pcdhb9*), the gene that showed the highest fold-change in mRNA expression levels in a comparison between wild-type and knockout mice, was tested with real-time PCR.

The primer sequences, product lengths, NCBI accession numbers and network classifications of the genes tested are shown in Table 1. From each sample, 1 µg of RNA was reverse transcribed using M-MLV reverse transcriptase (Promega, Mannheim, Germany), 500 ng oligo (dT)15 primer and 50 ng of a random primer mixture (Invitrogen, Solingen, Germany). Semiquantitative real-time RT–PCR was performed with the aid of QuantiTect SYBR Green master mix kit of Qiagen on the iCycler iQ Multicolor Real-Time PCR Detection System from Bio-Rad (Hercules, CA). All samples were analyzed in triplicate with a template amount corresponding to 2 ng of RNA. Hypoxanthine-phosphoribosyl-transferase (*HPRT*) was used as a housekeeping gene and all PCR products were subjected to automated sequencing to ensure amplification of the correct sequences.

**Table 1:** Description of primers.

**Gene**	**NCBI accession number**	**Forward primer (5′-3′)**	**Reverse primer (5′-3′)**	**Amplicon**	**Network**
*Akap9*	NM_194462.2	GTCTTCAGATGGTAGAAAAGGA	GTTAGCTGTGAGCTGAGTTATG	173 bp	A
*Setdb1*	NM_018877.2	GATAGCTCCTGCCGAGACTTC	CTGCCATCCACCTCTTCAAC	143 bp	A
*Snd1*	NM_019776.2	ACGCTGATGAGTTTGGCTACA	CCACGACAGAGGAGGTTTC	171 bp	A
*Crh*	NM_205769.1	CATTCTTGAGGGGTGGCTA	CTCTTACACAACCAAATTGACC	116 bp	B
*Igfbp3*	NM_008343.2	TCTTGGGGTCCTTCTCAAA	CCTCCAGACACAGGCTCC	194 bp	B
*Lasp1*	NM_010688.3	ATACCATGAGGAGTTTGAGAAG	ACCATAGGACGAGGTCATCT	196 bp	B
*Narf*	NM_026272.2	GATAGCATCCCTTCAGCCCT	TTCATCAAACCCCTTTATCTCC	156 bp	C
*Ogdh*	NM_010956.3	GCTAGTCTCTTCCTTGACTG	AACTTACTCATGCCATTGTC	184 bp	C
*Selenbp1*	NM_009150.3	GTGCAACGTGAGCAGTTT	CTGCATCCCCAGGCTTCT	161 bp	C
*Pcdhb9*	NM_053134.3	TTTAGGAGAAACTACCTTGTGC	TGAGCATTAAAGTCACTTGAGG	195 bp	none
*HRPT*	NM_013556.2	CCAGTAAAATTAGCAGGTGTTC	GATAAGCGACAATCTACCAGAG	179 bp	none

Shown are the NCBI accession numbers, primer sequences, product sizes and the assignment to the networks of the genes that were further tested with real-time RT–PCR.

### Statistics and data analysis

Data were analyzed using the software JMP 5.1 (SAS Institute, Cary, NC) and Excel (Microsoft Corporation, Redmond, WA). The mean cycle threshold (Ct) value of each triplet was taken and the mean normalized expression (MNE) was computed as previously described [33]. To test for differences between the four groups (wt/30, wt/42, hm/30, hm/42), ANOVA’s (ANOVA) were applied for each gene. In case the ANOVA was significant (p<0.05), a paired Student's *t*-test was applied as a post-hoc test to test for differences between wt/30 versus wt/42 and hm/30 versus hm/42.

## Results

### Microarray

#### Age-related comparisons (wt/30 versus wt/42) in wild-type mice

Comparing mRNA expression levels between the 30 days old and the 42 days old wild-type mice, 54 genes were classified as differentially expressed (with a minimum FC of ±1.5 and a p-value lower than 0.05). The corresponding genes are listed in Appendix 1 together with the fold-changes and p-values that were determined in homozygous Egr-1 knockout mice at the two ages. Seventeen genes showed reduced mRNA expression in the 42 days old wild-type mice compared to the 30 days old mice. Thirty-seven genes were higher expressed. The maximum fold-changes were −2.40 and 2.62, respectively.

### Age-related comparisons (hm/30 versus hm/42) in *Egr-1* knockout mice

Two hundred fifteen genes had changed their expression levels in the homozygous *Egr-1* knockout mice between the age of 30 and 42 days (see Appendix 2 for a list of those genes). Higher mRNA expression was found in 176 genes at 42 days, compared to 30 days, while 39 genes showed reduced mRNA expression. Age-dependent changes in gene expression ranged here between 2.49 fold and −4.01 fold. A pathway analysis was performed based on this list of differentially expressed genes. Genes that were further studied using semiquantitative real-time RT–PCR are shown in bold in Appendix 2.

Eight genes were differently expressed at the two ages in both wild-type and homozygous *Egr-1* knockout mice (shown in italics and underlined in Appendix 1 and Appendix 2). The directions of their changes were the same in wild-type- and homozygous Egr-1 knockout mice.

### *Egr-1*-related comparisons (wt/30 versus hm/30 and wt/42 versus hm/42)

In the 30 days old mice, the lack of Egr-1 was associated with different mRNA expression levels of 73 genes, with 39 upregulated and 34 downregulated (wt/30 versus hm/30, see Table 2 for a list of those genes). In the 42 days old mice, 135 genes were differently expressed compared to the wild-type. One hundred and thirteen genes were upregulated, and 22 genes were downregulated (wt/42 versus hm/42, see Table 3 for a list of those genes).

**Table 2:** List of genes that were differentially expressed between wild-type and homozygous *Egr-1* knockout mice at the age of 30 days (wt/30 versus hm/30).

**Affymetrix ID**	**Gene symbol**	**Gene title**	**FC (wt/p30 vs hm/p30)**	**p-value (wt/p30 vs hm/p30)**	**FC (wt/p42 vs hm/p42)**	**p-value (wt/p42 vs hm/p42)**
**Translation factor activity (1)**						
1438686_at	Eif4g1	eukaryotic translation initiation factor 4, gamma 1	−1.71	0.0207	−1.15	0.1370
**Electron Carrier Activity (1)**						
1417590_at	Cyp27a1	cytochrome P450, family 27, subfamily a, polypeptide 1	1.54	0.0047	−1.09	0.5424
**Structural molecule activity (2)**						
1418306_at	Crybb1	crystallin, beta B1	−1.98	0.0291	−1.40	0.5518
1419011_at	Cryba2	crystallin, beta A2	−2.02	0.0236	−1.92	0.3953
**Enzyme Regulator Activity (3)**						
1422477_at	Cables1	Cdk5 and Abl enzyme substrate 1	1.57	0.0067	1.03	0.8213
1423062_at	Igfbp3	insulin-like growth factor binding protein 3	2.05	0.0281	−1.09	0.6077
1421138_a_at	Pkib	protein kinase inhibitor beta	−1.63	0.0356	−1.16	0.2212
**Transcription Regulator Activity (3)**						
1445710_x_at	Duxbl	double homeobox B-like	1.82	0.0010	1.19	0.4352
*1417065_at*	*Egr1*	*early growth response 1*	*1.99*	*0.0000*	*1.79*	*0.0004*
*1417930_at*	*Nab2*	*Ngfi-A binding protein 2*	*−2.40*	*0.0035*	*−2.88*	*0.0001*
**Molecular Transducer Activity (4)**						
1428538_s_at	Rarres2	retinoic acid receptor responder (tazarotene induced) 2	1.52	0.0401	−1.02	0.9101
1430295_at	Gna13	guanine nucleotide binding protein, alpha 13	1.68	0.0012	1.08	0.4900
1434447_at	Met	met proto-oncogene	1.69	0.0380	−1.17	0.1753
1418552_at	Opn1sw	opsin 1 (cone pigments), short-wave-sensitive	−1.72	0.0000	−1.40	0.0233
**Transporter Activity (5)**						
1424338_at	Slc6a13	solute carrier family 6, member 13	1.66	0.0068	−1.37	0.1628
1443823_s_at	Atp1a2	ATPase, Na^+^/K+ transporting, alpha 2 polypeptide	1.76	0.0136	−1.09	0.6566
1438945_x_at	Gja1	gap junction membrane channel protein alpha 1	1.83	0.0229	−1.14	0.5575
*1436044_at*	*Scn7a*	*sodium channel, voltage-gated, type VII, alpha*	*−1.59*	*0.0005*	*−1.74*	*0.0000*
*1415844_at*	*Syt4*	*synaptotagmin IV*	*−2.55*	*0.0000*	*−2.26*	*0.0000*
**Catalytic Activity (20)**						
1452839_at	Dph5	DPH5 homolog (S. cerevisiae)	1.51	0.0197	1.25	0.0094
1440926_at	Flt1	FMS-like tyrosine kinase 1	1.56	0.0263	−1.18	0.2562
1428987_at	Dynlrb2	dynein light chain roadblock-type 2	1.62	0.0199	1.49	0.2117
1449623_at	Txnrd3	thioredoxin reductase 3	1.64	0.0080	1.07	0.6259
*1417024_at*	*Hars*	*histidyl-tRNA synthetase*	*1.66*	*0.0000*	*1.66*	*0.0000*
1440179_x_at	Ibrdc1	IBR domain containing 1	1.70	0.0067	−1.10	0.1262
1455385_at	Exoc6	exocyst complex component 6	1.76	0.0093	1.09	0.6328
1454713_s_at	Hdc	histidine decarboxylase	1.80	0.0150	1.21	0.0278
1449106_at	Gpx3	glutathione peroxidase 3	1.93	0.0472	−1.80	0.2441
1452975_at	Agxt2l1	alanine-glyoxylate aminotransferase 2-like 1	2.29	0.0072	−1.24	0.1606
1424325_at	Esco1	establishment of cohesion 1 homolog 1 (S. cerevisiae)	−1.53	0.0239	−1.22	0.1796
1430996_at	Etnk1	ethanolamine kinase 1	−1.53	0.0076	−1.13	0.4668
1441486_at	Fkbp15	FK506 binding protein 15	−1.54	0.0040	−1.38	0.1085
1434734_at	E130016E03Rik	RIKEN cDNA E130016E03 gene	−1.57	0.0446	−1.00	0.9955
1458363_at	Zdhhc17	zinc finger, DHHC domain containing 17	−1.58	0.0012	−1.25	0.0814
1440553_at	Mecr	mitochondrial trans-2-enoyl-CoA reductase	−1.58	0.0262	−1.16	0.1769
1445632_at	Ogdh	oxoglutarate dehydrogenase (lipoamide)	−1.58	0.0050	1.09	0.6322
1440351_at	Birc4	Baculoviral IAP repeat-containing 4	−1.75	0.0283	1.02	0.8804
1457732_at	Pcmtd2	Protein-L-isoaspartate O-methyltransferase domain containing 2	−2.00	0.0016	−1.01	0.9495
*1439843_at*	*Camk4*	*calcium/calmodulin-dependent protein kinase IV*	*−2.19*	*0.0000*	*−1.95*	*0.0006*
**Binding (24)**						
1438295_at	Glcci1	Glucocorticoid induced transcript 1	1.50	0.0044	1.21	0.2239
1441317_x_at	Jakmip1	janus kinase and microtubule interacting protein 1	1.50	0.0256	1.04	0.5407
1434203_at	Fam107a	Fam107a family with sequence similarity 107, member A	1.52	0.0244	−1.07	0.4713
1451602_at	Snx6	sorting nexin 6	1.55	0.0456	1.06	0.6713
1428942_at	Mt2	metallothionein 2	1.57	0.0008	−1.30	0.0078
1435386_at	Vwf	Von Willebrand factor homolog	1.60	0.0158	1.31	0.0166
1452217_at	Ahnak	AHNAK nucleoprotein (desmoyokin)	1.61	0.0073	−1.01	0.9348
1422660_at	LOC671237	similar to Putative RNA-binding protein 3	1.62	0.0121	−1.04	0.6603
*1456351_at*	*Brd8*	*bromodomain containing 8*	*1.67*	*0.0003*	*1.66*	*0.0005*
*1429239_a_at*	*Stard4*	*StAR-related lipid transfer (START) domain containing 4*	*2.18*	*0.0020*	*1.79*	*0.0295*
1417580_s_at	Selenbp1	selenium binding protein 1	2.34	0.0102	1.23	0.0676
*1451692_at*	*Tmco6*	*transmembrane and coiled-coil domains 6*	*4.10*	*0.0000*	*2.73*	*0.0002*
1422877_at	Pcdhb12	protocadherin beta 12	−1.52	0.0070	−1.41	0.0010
1437694_at	BB114266	Expressed sequence BB114266	−1.53	0.0318	−1.13	0.1606
1421132_at	Pvrl3	poliovirus receptor-related 3	−1.56	0.0153	1.02	0.7130
1436981_a_at	Ywhaz	tyrosine 3-monooxygenase activation protein, zeta	−1.56	0.0024	−1.04	0.5712
1440632_at	Pcdhb4	protocadherin beta 4	−1.57	0.0446	−1.58	0.0833
*1449527_at*	*Pcdhb7*	*protocadherin beta 7*	*−1.58*	*0.0155*	*−1.64*	*0.0016*
1430569_at	Ttc9c	tetratricopeptide repeat domain 9C	−1.58	0.0089	−1.02	0.8478
1443950_at	A630042L21Rik	RIKEN cDNA A630042L21 gene	−1.64	0.0345	1.33	0.0600
1421953_at	Crkl	v-crk sarcoma virus CT10 oncogene homolog (avian)-like	−1.66	0.0216	1.01	0.9614
1426458_at	Slmap	sarcolemma associated protein	−1.68	0.0164	−1.39	0.1939
*1443315_at*	*Dmd*	*Dystrophin*	*−2.45*	*0.0492*	*1.66*	*0.0103*
**1422640_at**	**Pcdhb9**	**protocadherin beta 9**	**−14.34**	**0.0000**	**−17.48**	**0.0000**
**Unknown (8)**						
1417460_at	Ifitm2	interferon induced transmembrane protein 2	1.53	0.0478	−1.03	0.7015
1417275_at	Mal	myelin and lymphocyte protein, T-cell differentiation protein	1.59	0.0059	1.16	0.1316
1453632_at	4930538K18Rik	RIKEN cDNA 4930538K18 gene	1.61	0.0215	1.07	0.5032
1434817_s_at	Rprd2	Regulation of nuclear pre-mRNA domain containing 2	1.75	0.0387	1.13	0.2222
1460049_s_at	1500015O10Rik	RIKEN cDNA 1500015O10 gene	1.77	0.0163	1.34	0.3090
1447553_x_at	Ric3	Resistance to inhibitors of cholinesterase 3 homolog (C. elegans)	−1.56	0.0314	1.33	0.1653
1451634_at	2810051F02Rik	RIKEN cDNA 2810051F02 gene	−1.63	0.0438	1.107	0.5203
*1428909_at*	*A130040M12Rik*	*RIKEN cDNA A130040M12 gene*	*−1.90*	*0.0001*	*−1.62*	*0.0368*
**Not Annotated (2)**						
1441430_at			1.56	0.0106	−1.13	0.2617
1442733_at			−1.55	0.0244	−1.05	0.7378

Shown are Affymetrix ID, gene symbol, gene title, fold change (FC) and p-values that indicate the differences in mRNA expression levels between the wild-type and the homozygous *Egr-1* knockout mice both at the age of 30 days and 42 days. Genes were sorted after GO annotations. Genes that were significantly changed (with a FC > 1.5 and p-value < 0.05) in both the 30 days old mice and the 42 days old mice are shown in italics and are underlined. The gene that showed the highest differences in mRNA expression levels between the homozygous and the wild-type mice (*Pcdhb9* (shown in bold)) was further investigated using real-time PCR.

**Table 3:** List of genes that were differentially expressed between wild-type and homozygous *Egr-1* knockout mice at the age of 42 days (wt/42 versus hm/42).

**Affymetrix ID**	**Gene symbol**	**Gene title**	**FC (wt/p42 vs hm/p42)**	**p-value (wt/p42 vs hm/p42)**	**FC (wt/p30 vs hm/p30)**	**p-value (wt/p30 vs hm/p30)**
**Structural Molecule Activity (1)**						
1421811_at	Thbs1	thrombospondin 1	−2.09	0.0195	1.06	0.7630
**Transporter Activity (4)**						
1458916_at	Slc12a6	Solute carrier family 12, member 6	1.61	0.0076	−1.08	0.6697
1457497_at	Syt1	Synaptotagmin I	1.64	0.0052	−1.16	0.6213
*1436044_at*	*Scn7a*	*sodium channel, voltage-gated, type VII, alpha*	*−1.89*	*0.0003*	*−1.28*	*0.0383*
*1415844_at*	*Syt4*	*synaptotagmin IV*	*−2.26*	*0.0000*	*−2.55*	*0.0000*
**Enzyme Regulator Activity (7)**						
1444671_at	Rasal2	RAS protein activator like 2	1.53	0.0363	−1.30	0.2962
1446595_at	Itsn2	intersectin 2	1.53	0.0182	−1.26	0.4784
1440347_at	Arhgap10	Rho GTPase activating protein 10	1.57	0.0115	1.02	0.9324
1441386_at	Rapgef1	Rap guanine nucleotide exchange factor 1	1.61	0.0194	−1.12	0.1515
1445307_at	Auts2	Autism susceptibility candidate 2	1.86	0.0009	−1.13	0.7187
1442897_at	2610024E20Rik	RIKEN cDNA 2610024E20 gene	−1.50	0.0219	−1.39	0.0095
1416188_at	Gm2a	GM2 ganglioside activator protein	−1.79	0.0475	−1.02	0.8384
**Molecular Transducer Activity (8)**						
1458469_at	Cblb	Casitas B-lineage lymphoma b	1.61	0.0089	−1.13	0.6019
1455967_at	Sorbs1	sorbin and SH3 domain containing 1	1.58	0.0111	1.10	0.7148
1445555_at	Trpm3	Transient receptor potential cation channel, subfamily M, member 3	1.64	0.0456	−1.13	0.5703
1443279_at	Nlk	Nemo like kinase	1.55	0.0307	−1.09	0.7229
1441498_at	Ptprd	Protein tyrosine phosphatase, receptor type, D	1.57	0.0499	1.12	0.8303
1441220_at	Magi2	Membrane associated guanylate kinase, WW and PDZ domain containing 2	1.93	0.0316	1.10	0.8184
1422723_at	Stra6	stimulated by retinoic acid gene 6	−1.59	0.0205	1.22	0.3171
1417205_at	Kdelr2	KDEL (Lys-Asp-Glu-Leu) endoplasmic reticulum protein retention receptor 2	−1.53	0.0027	1.14	0.3870
**Transcription Regulator Activity (13)**						
1441615_at	Cbfa2t2	core-binding factor, runt domain, alpha subunit 2, translocated to, 2 (human)	1.53	0.0280	−1.02	0.92091
1445914_at	Nrf1	Nuclear respiratory factor 1	1.543	0.0016	1.31	0.5977
1441140_at	Rere	Arginine glutamic acid dipeptide (RE) repeats	1.55	0.0021	1.27	0.3524
1456686_at	Zfhx1a	Zinc finger homeobox 1a	1.56	0.0159	1.04	0.7871
1439946_at	Mef2c	Myocyte enhancer factor 2C	1.62	0.0041	−1.14	0.5653
1458661_at	Lcor	Ligand dependent nuclear receptor corepressor	1.63	0.0265	−1.04	0.9105
1446953_at	Tcf4	Transcription factor 4	1.63	0.0097	1.03	0.9567
1445695_at	Atxn1	Ataxin 1	1.73	0.0110	−1.40	0.3692
*1417065_at*	*Egr1*	*early growth response 1*	*1.79*	*0.0004*	*1.99*	*0.0000*
1443511_at	Rora	RAR-related orphan receptor alpha	1.96	0.0294	−1.18	0.5929
1436329_at	Egr3	early growth response 3	−1.51	0.0326	−1.30	0.1831
1443897_at	Ddit3	DNA-damage inducible transcript 3	−1.55	0.0297	1.11	0.6698
*1417930_at*	*Nab2*	*Ngfi-A binding protein 2*	*−2.88*	*0.0001*	*−2.40*	*0.0035*
**Catalytic Activity (17)**						
1458663_at	Large	Like-glycosyltransferase	1.50	0.0298	1.37	0.2474
1442813_at	Dgki	Diacylglycerol kinase, iota	1.53	0.0448	−1.00	0.9982
1435273_at	Wars2	tryptophanyl tRNA synthetase 2 (mitochondrial)	1.53	0.0008	1.10	0.6011
1443445_at	Diap3	Diaphanous homolog 3 (Drosophila)	1.61	0.0162	−1.32	0.0682
1442163_at	Hace1	HECT domain and ankyrin repeat containing, E3 ubiquitin protein ligase 1	1.64	0.0043	−1.11	0.7030
*1417024_at*	*Hars*	*histidyl-tRNA synthetase*	*1.66*	*0.0000*	*1.66*	*0.0000*
1440066_at	Smarcad1	SWI/SNF-related, matrix-associated actin-dependent regulator of chromatin, subfamily a	1.69	0.0420	1.16	0.1397
1445395_at	Prkca	Protein kinase C, alpha	1.69	0.0030	1.04	0.9369
1438583_at	Ern1	Endoplasmic reticulum (ER) to nucleus signaling 1	1.71	0.0264	−1.50	0.0969
1446412_at	Wwox	WW domain-containing oxidoreductase	1.76	0.0152	1.104	0.6550
1445188_at	Gphn	Gephyrin	1.80	0.0010	−1.13	0.7227
1455905_at	2610507B11Rik	RIKEN cDNA 2610507B11 gene	−1.50	0.0435	−1.15	0.3423
1430177_at	Ube2b	ubiquitin-conjugating enzyme E2B, RAD6 homology (S. cerevisiae)	−1.52	0.0099	−1.33	0.2071
1439540_at	March2	membrane-associated ring finger (C3HC4) 2	−1.52	0.0115	−1.40	0.0493
1416613_at	Cyp1b1	cytochrome P450, family 1, subfamily b, polypeptide 1	−1.54	0.0402	−1.19	0.3717
1421024_at	Agpat1	1-acylglycerol-3-phosphate O-acyltransferase 1	−1.55	0.0048	1.02	0.8328
*1439843_at*	*Camk4*	*calcium/calmodulin-dependent protein kinase IV*	*−1.95*	*0.0006*	*−2.19*	*0.0000*
**Binding (30)**						
1441567_at	Myo9a	Myosin IXa	1.50	0.0249	1.07	0.8016
1447381_at	Cpsf6	Cleavage and polyadenylation specific factor 6	1.51	0.0298	1.12	0.5466
1456773_at	Nupl2	nucleoporin like 2	1.51	0.0362	−1.07	0.6814
1441373_at	Msi2	Musashi homolog 2 (Drosophila)	1.53	0.0046	1.14	0.6634
1436382_at	Zbtb12	zinc finger and BTB domain containing 12	1.54	0.0359	−1.18	0.1262
1456303_at	Phf14	PHD finger protein 14	1.54	0.0178	1.07	0.8732
1443199_at	Lrch3	Leucine-rich repeats and calponin homology (CH) domain containing 3	1.55	0.0347	−1.29	0.2964
1443337_at	Grip1	Glutamate receptor interacting protein 1	1.55	0.0332	−1.30	0.5147
1447615_at	Fmn1	Formin 1	1.55	0.0082	−1.19	0.6470
1455893_at	Rspo2	R-spondin 2 homolog (*Xenopus laevis*)	1.55	0.0419	1.09	0.5464
1446279_at	Negr1	Neuronal growth regulator 1	1.58	0.0314	1.02	0.9288
1445329_at	Dtnb	dystrobrevin, beta	1.58	0.0214	−1.07	0.7944
1444384_at	Jazf1	JAZF zinc finger 1	1.58	0.0358	−1.34	0.2980
1439123_at	Phf21a	PHD finger protein 21A	1.60	0.0102	−1.14	0.6756
1441769_at	Arl15	ADP-ribosylation factor-like 15	1.61	0.0200	−1.03	0.9351
1442411_at	Glcci1	Glucocorticoid induced transcript 1	1.63	0.0068	−1.16	0.6206
1440551_at	Dnajc1	DnaJ (Hsp40) homolog, subfamily C, member 1	1.66	0.0280	1.21	0.5996
1440543_at	D930036F22Rik	RIKEN cDNA D930036F22 gene	1.66	0.0428	−1.02	0.9482
*1443315_at*	*Dmd*	*Dystrophin*	*1.66*	*0.0103*	*−2.45*	*0.0492*
*1456351_at*	*Brd8*	*bromodomain containing 8*	*1.66*	*0.0005*	*1.67*	*0.0003*
1446481_at	Apbb2	Amyloid beta (A4) precursor protein-binding, family B, member 2	1.68	0.0279	1.06	0.8385
1458263_at	Cugbp2	CUG triplet repeat, RNA binding protein 2	1.74	0.0041	−1.10	0.8176
1440067_at	Ncam1	Neural cell adhesion molecule 1	1.75	0.0171	1.03	0.9102
1444488_at	Cadps	Ca<2+>dependent activator protein for secretion	1.76	0.0054	1.01	0.9801
1441330_at	Crb1	crumbs homolog 1 (Drosophila)	1.99	0.0214	−1.12	0.4163
*1429239_a_at*	*Stard4*	*StAR-related lipid transfer (START) domain containing 4*	*2.18*	*0.001991*	*1.79*	*0.0295*
*1451692_at*	*Tmco6*	*transmembrane and coiled-coil domains 6*	*2.73*	*0.0002*	*4.10*	*0.000*
1449548_at	Efnb2	ephrin B2	−1.58	0.0436	−1.05	0.8291
*1449527_at*	*Pcdhb7*	*protocadherin beta 7*	*−1.64*	*0.0016*	*−1.58*	*0.0155*
**1422640_at**	**Pcdhb9**	**protocadherin beta 9**	**−17.48**	**0.0000**	**−14.34**	**0.0000**
**Unknown (43)**						
1454397_at	4632418H02Rik	RIKEN cDNA 4632418H02 gene	1.50	0.0066	−1.05	0.8725
1432713_at	6430709C05Rik	RIKEN cDNA 6430709C05 gene	1.51	0.0031	−1.05	0.8923
1442509_at	Evi5	Ecotropic viral integration site 5	1.52	0.0156	−1.03	0.9005
1429900_at	5330406M23Rik	RIKEN cDNA 5330406M23 gene	1.52	0.0119	1.12	0.8002
1444651_at	LOC553089	hypothetical LOC553089	1.53	0.0230	−1.42	0.3710
1459409_at	Ccdc109a	coiled-coil domain containing 109A	1.53	0.0337	1.01	0.9616
1440570_at	Lhfpl3	Lipoma HMGIC fusion partner-like 3	1.53	0.010572	1.29	0.4440
1430195_at	2810043O03Rik	RIKEN cDNA 2810043O03 gene	1.54	0.0153	−1.16	0.7564
1438788_at	D5Wsu152e	DNA segment, Chr 5, Wayne State University 152	1.54	0.0060	1.15	0.7128
1444137_at	A430108G06Rik	RIKEN cDNA A430108G06 gene	1.54	0.0017	1.03	0.8671
1457781_at	Kcnq1ot1	KCNQ1 overlapping transcript 1	1.54	0.0013	−1.00	0.9993
1430096_at	2900017F05Rik	RIKEN cDNA 2900017F05 gene	1.56	0.0160	1.14	0.5974
1443088_at	9930031P18Rik	RIKEN cDNA 9930031P18 gene	1.56	0.0026	1.16	0.7326
1458706_at	2610035D17Rik	RIKEN cDNA 2610035D17 gene	1.56	0.0048	−1.08	0.5992
1453706_at	2900042A17Rik	RIKEN cDNA 2900042A17 gene	1.57	0.0272	1.02	0.9373
1443489_at	Vps13b	Vacuolar protein sorting 13B (yeast)	1.57	0.0012	1.01	0.9575
1440604_at	8030494B02Rik	Riken cDNA 8030494B02 gene	1.57	0.0255	−1.35	0.2507
1444445_at	C77648	expressed sequence C77648	1.57	0.0475	−1.10	0.6343
1433837_at	8430408G22Rik	RIKEN cDNA 8430408G22 gene	1.57	0.0226	−1.00	0.9943
1457508_at	C430003N24Rik	RIKEN cDNA C430003N24 gene	1.60	0.0022	−1.34	0.3974
1454558_at	5430416B10Rik	RIKEN cDNA 5430416B10 gene	1.60	0.0256	−1.07	0.8665
1440892_at	BC017647	CDNA sequence BC017647	1.63	0.0403	−1.06	0.8572
1441467_at	Tspan5	Tetraspanin 5	1.64	0.0438	−1.10	0.6658
1460101_at	NRXN3	Neurexin 3	1.67	0.0353	−1.05	0.9144
1444109_at	C130009A20Rik	RIKEN cDNA C130009A20 gene	1.67	0.0109	1.01	0.9683
1454424_at	2610040L17Rik	RIKEN cDNA 2610040L17 gene	1.68	0.0058	1.06	0.8692
1433266_at	2810416A17Rik	RIKEN cDNA 2810416A17 gene	1.66	0.0005	−1.14	0.7660
1442561_at	Mamdc1	MAM domain containing 1	1.69	0.0202	−1.06	0.9040
1453906_at	Med13l	mediator complex subunit 13-like	1.69	0.0010	1.02	0.9415
1443201_at	Gpc6	Glypican 6	1.70	0.0288	−1.20	0.6594
1458309_at	Dip2c	disco-interacting protein 2 homolog C	1.73	0.0250	−1.04	0.8794
1458505_at	LOC552901	hypothetical LOC552901	1.75	0.0206	−1.20	0.5693
1429977_at	9030425L15Rik	RIKEN cDNA 9030425L15 gene	1.76	0.0068	−1.07	0.9202
1440513_at	C80258	expressed sequence C80258	1.79	0.0100	−1.20	0.6384
1429870_at	C630040K21Rik	RIKEN cDNA C630040K21 gene	1.79	0.0029	−1.01	0.9797
1446102_at	D9Ertd292e	DNA segment, Chr 9, ERATO Doi 292, expressed	1.81	0.0028	−1.23	0.2711
1458779_at	8030445P17Rik	RIKEN cDNA 8030445P17 gene	1.83	0.0154	−1.17	0.763599
1453897_at	C030014A21Rik	RIKEN cDNA C030014A21 gene	1.85	0.0025	−1.46	0.265201
1446606_at	LOC625175	hypothetical protein A630054D14	1.86	0.0062	1.30	0.187229
1432757_at	2900011L18Rik	RIKEN cDNA 2900011L18 gene	1.90	0.0232	1.30	0.623096
1441231_at	LOC100042016	hypothetical protein LOC100042016	1.92	0.0002	−1.06	0.840764
1439086_at	A930009L07Rik	RIKEN cDNA A930009L07 gene	−1.58	0.0294	1.19	0.34015
*1428909_at*	*A130040M12Rik*	*RIKEN cDNA A130040M12 gene*	*−1.62*	*0.0368*	*−1.90*	*0.0001*
**Not Annotated (12)**						
1446799_at			1.50	0.0030	−1.10	0.8224
1443271_at			1.51	0.0384	−1.46	0.3101
1441740_at			1.52	0.0284	−1.22	0.5593
1443526_at			1.52	0.0062	1.03	0.9444
1458077_at			1.55	0.0148	−1.01	0.9758
1445740_at			1.56	0.0456	−1.00	0.9998
1435409_at			1.61	0.0220	1.59	0.0878
1439999_at			1.63	0.0223	−1.11	0.7133
1443744_at			1.72	0.0128	−1.14	0.7148
1444622_at			1.82	0.0224	−1.02	0.9539
1457479_at			1.95	0.0060	−1.00	0.9815
1459595_at			2.18	0.0019	1.07	0.8169

Shown are Affymetrix ID, gene symbol, gene title, fold change (FC) and p-values that indicate the differences in gene expression levels between the wild-type and the homozygous *Egr-1* knockout mice both at the age of 42 days and 30 days. Genes were sorted after GO annotations. Genes that were significantly changed (with a FC > 1.5 and p-value < 0.05) in both the 42 days old mice and the 30 days old mice are shown in italics and are underlined. The gene that showed the highest differences in mRNA expression levels between the homozygous and the wild-type mice (*Pcdhb9* [shown in bold]) was further investigated using real-time PCR.

Thirteen genes showed up in both lists (shown in italics and underlined in both Table 2 and Table 3) and except for one gene (dystrophin (*DMD*)), the regulation of mRNA expression levels was in the same direction at both 30 days and 42 days. The gene that showed the highest difference in mRNA expression levels (*Pcdhb9,* shown in bold in Table 2 and Table 3), was further studied using semiquantitative real-time RT–PCR.

### Pathway analyses

Data obtained by comparison of the mRNA expression patterns in the homozygous *Egr-1* knockout mice between the age of 30 days and 42 days (hm/30 versus hm/42, see Appendix 2, 215 genes) were analyzed using the software “Ingenuity Pathway Analysis.” Several networks were identified that could be involved in retinal signaling. We chose three networks that appeared different in the homozygous *Egr-1* knockout mice (see Figure 1A-C) to find candidates for validation by real-time RT–PCR. Networks proposed by the software were common signaling pathways. All genes that were found to be differently expressed in the knockout mice are indicated by filled gray symbols in Figure 1. Genes labeled as open symbols represent intermediate metabolic steps, determined from the current literature by the software. Molecules represented in network A (Figure 1A) are regulated by mitogen-activated protein kinases (MAPK), which respond to a variety of extracellular stimuli and regulate various cellular activities, such as gene expression, mitosis, differentiation, and cell survival/apoptosis [34]. These kinases seem to be effector proteins in network B (Figure 1B) as well, together with insulin, the early response transcription factor activator-protein 1 (Ap1) and another ubiquitous transcription factor, nuclear factor-kappa B (NfkB). The central molecules in network C (Figure 1C) are retinoic acid, vascular endothelial growth factor A (Vegf-A), V-fos FBJ murine osteosarcoma viral oncogene homolog (Fos) and beta-estradiol.

Furthermore, several pathways were identified in both the wild-type and the homozygous knockout mice (wt/30 versus wt/42 and hm/30 versus hm/42, shown in Table 4). The lack of Egr-1 seems to affect a variety of pathways and many fundamental pathways are part of this list (e.g., synaptic long-term depression and potentiation, PDGF- and chemokine signaling, and others).

**Table 4:** Pathways identified in the wild-type and the homozygous *Egr-1* knockout mice by Ingenuity Pathway Analysis Software (wt/30 versus wt/42 and hm/30 versus hm/42).

**Pathways detected in wild-type mice (30 days versus 42 days, 54 differentially expressed genes):**	
**Pathway**	**Molecules**
Arachidonic Acid Metabolism	*Gpx3, Cyp2d6, Ptgds*
Acute Phase Response Signaling	*Ttr, Tf, A2m*
FXR/RXR Activation	*Pon1, Cyp27a1*
**Pathways detected in Egr-1 knockout-mice (30 days versus 42 days, 215 differentially expressed genes):**	
**Pathway**	**Molecules**
Synaptic Long-term Depression	*Plcb4, Itpr2, Igf1r, Gna13, Prkca, Prkcb1*
Synaptic Long-term Potentiation	*Plcb4, Itpr2, Atf2, Prkca, Prkcb1*
Huntington's Disease Signaling	*Gnb1, Plcb4, Igf1r, Zdhhc17, Atf2, Prkca, Prkcb1*
PDGF Signaling	*Crkl, Abl1, Prkca, Prkcb1*
Chemokine Signaling	*Plcb4, Ppp1r12b, Prkca, Prkcb1*
VDR/RXR Activation	*Igfbp3, Ncoa1, Prkca, Prkcb1*
FGF Signaling	*Crkl, Fgf11, Atf2, Prkca*
Ephrin Receptor Signaling	*Gnb1, Crkl, Abl1, Gna13, Atf2, Rapgef1*
Axonal Guidance Signaling	*Gnb1, Plcb4, Pfn1, Crkl, Abl1, Gna13, Prkca, Prkcb1*
Lysine Degradation	*Setdb1, Nsd1, Ogdh*
Actin Cytoskeleton Signaling	*Pfn1, Diaph3, Crkl, Ppp1r12b, Fgf11, Gna13*
Circadian Rhythm Signaling	*Arntl, Atf2*
G-Protein Coupled Receptor Signaling	*Pde8a, Plcb4, Atf2, Prkca, Prkcb1*
Integrin Signaling	*Tspan5, Crkl, Ppp1r12b, Abl1, Rapgef1*
Neuregulin Signaling	*Crkl, Prkca, Prkcb1*
SAPK/JNK Signaling	*Gnb1, Crkl, Gna13, Atf2*
ERK/MAPK Signaling	*Crkl, Atf2, Rapgef1, Prkca, Prkcb1*

Shown are the descriptions of the pathways and the molecules involved in both the wild-type and the homozygous *Egr-1* knockout mice that changed their mRNA expression patterns over the investigated period of time (p30 versus p42) based on Ingenuity Pathway Analysis Software.

### Real-time RT–PCR

**Figure 2:**
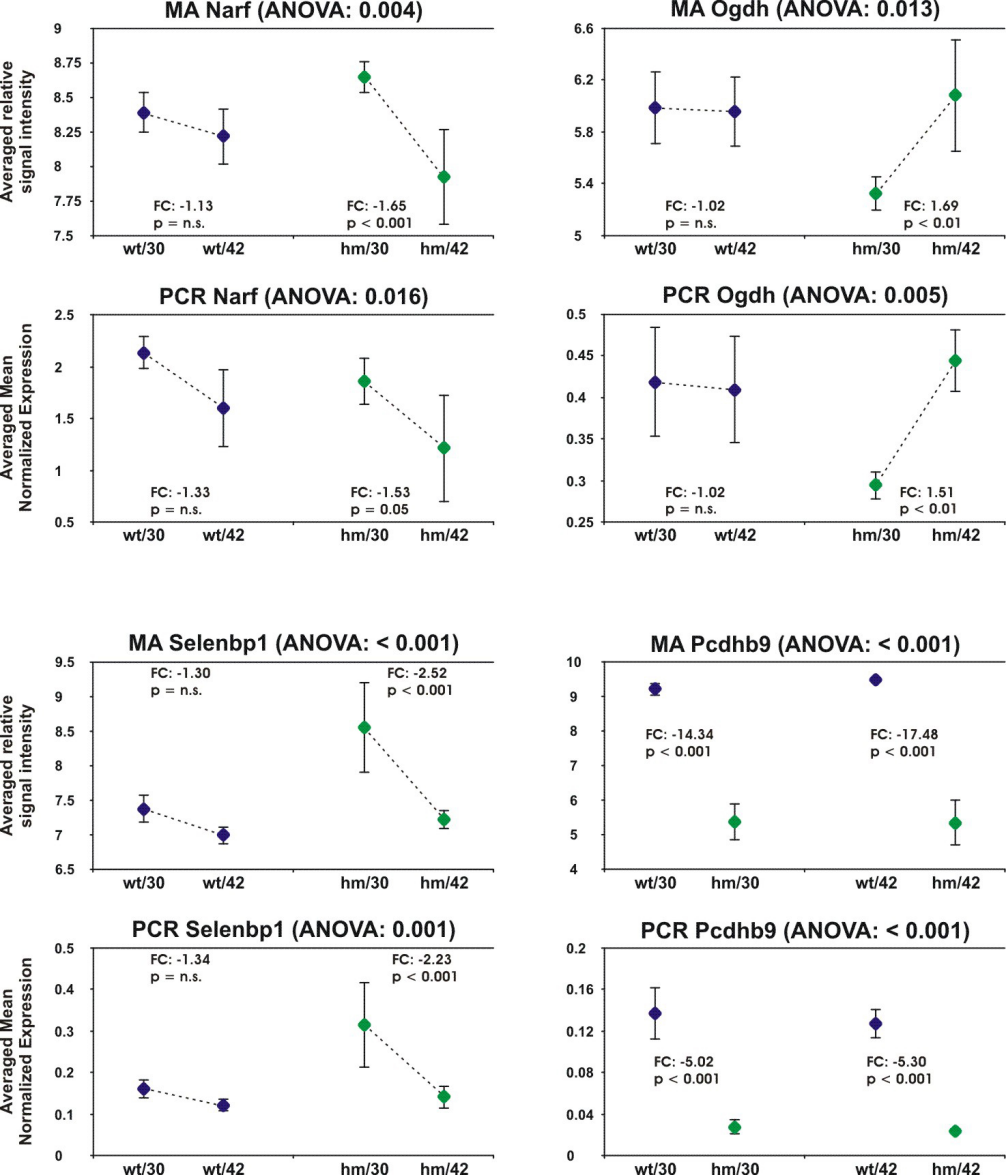
Genes whose mRNA expression levels could be validated by real-time RT–PCR. Mean relative expression values ±SD (n=4) obtained by microarray analysis and mean normalized expression values obtained by real-time PCR (n=4). The p-values of the ANOVA's can be seen in the heading of the figures. The fold-changes and p-values (determined by un-paired Student's *t*-test as post-hoc analysis) were computed for the comparison between 30 days and 42 days old knockout- and wild-type mice (wt/30 versus wt/42 and hm/30 versus. hm/42) and are shown within the figures. Please note that in the case of *Pcdhb9*, the comparison is between the knockout and the wild-type mice at both ages (wt/30 versus. hm/30 and wt42 versus hm/42).

Based on the three networks identified by Ingenuity Pathways Analysis Software (hm/30 versus hm/42, Figure 1A-C), nine genes were chosen for validation of their changes in mRNA expression by real-time RT–PCR, with three genes present in each network. In addition, protocadherin-beta 9 (*Pcdhb9*), the gene that showed the highest fold-change in comparison between the wild-type and the knockout mice (wt/30 versus hm/30: 14-fold lower expressed in the homozygous mice and wt/42 versus hm/42: 17-fold lower expressed) was tested with real-time RT–PCR. The changes in mRNA expression levels of four of the genes could be validated (Figure 2). Three of them were chosen based on network C (*Narf*, *Ogdh*, and *Selenbp1*) and the gene which showed the biggest difference in mRNA expression levels between wild-type and knockout mice, *Pcdhb9*, was also confirmed.

The relative signal intensities obtained by microarray analysis (GC-RMA based, shown are the log transformed mean relative signal intensities ±SD, n=4 each) of all four groups tested are shown in Figure 2, upper graphs. The lower graphs shows the mean MNE-values ±SD obtained by real-time RT–PCR (n=4 each). The results of the ANOVA is indicated in the header of the figures for each gene and the fold-changes (FC) and p values (p) of the un-paired Student's *t*-tests as post-hoc tests for the comparisons between the 30 days old mice and the 42 days old mice are shown within the graphs.

Note that the comparison of the *Pcdhb9* expression is not between the two age groups, but between homozygous knockout mice and wild-type mice at both time points.

Nuclear prelamin A recognition factor (*Narf*) was lower expressed in the 42 days old homozygous mice, compared to the 30 days old mice in both the microarray analysis (hm/42 versus hm/30: −1.65 fold, p<0.001) and the real-time PCR experiment (−1.53 fold, p<0.05). The mRNA expression did not differ in the wild-type mice over time in either experiment.

Also Oxoglutarate dehydrogenase (*Ogdh*) was different only in the homozygous *Egr-1* knockout mice and showed no changes in mRNA expression levels over time in the wild-type. Post-hoc analysis confirmed that mRNA expression levels in the 30 day-old homozygous mice was significantly lower, compared to 42 day-old animals (hm/30 versus hm/42: 1.69-fold, with p<0.01 in the MA experiment and 1.51 fold with p<0.01 in the PCR experiment).

The mRNA expression levels of selenium binding protein 1 (*Selenbp1*) were higher in the 30 days old knockout mice, compared to the 42 days old homozygous mice (hm/42 versus hm/30: −2.52 fold with p<0.001 in the MA experiment and −2.23 fold with p<0.001 in the PCR experiment) and again, no such changes were observed in the wild-type.

In case of protocadherin-beta 9 (*Pcdhb9*), mRNA expression levels did not change with age, but were much lower in the knockout mice. Real-time PCR detected a fivefold decline in the expression of *Pcdhb9* in knockout mice, both at p30 and at p42, whereas the microarray analysis determined an even larger decline (hm/30 versus wt/30: −14 fold and hm/42 versus wt/42: −17 fold, with p<0.001 each).

## Discussion

Earlier studies suggested that *Egr-1* might play a role in eye growth regulation in chicks [13] and mice [20,35]. The current study attempted to determine which genes were differentially regulated in Egr-1 knockout mice at two different developmental stages. Since *Egr-1* knockout mice had longer eyes at age 42 days, compared to wild-type mice, but not at day 30, it was hoped that a correlation could be found between the expression of certain genes and changes in eye growth. Furthermore, it was hoped that some of the changes might occur that relate to genes or factors that are already known to be involved in the regulation of axial eye growth in animal models.

The analysis of retinal gene expression in homozygous *Egr-1* knockout mice and wild-type mice at different ages (p30 and p42) provided a huge amount of data. Depending on the comparisons (wild-type versus homozygous, 30 days versus 42 days), different information about gene expression were obtained and different conclusions could be drawn. The focus in this paper was on changes that occur in homozygous *Egr-1* knockout mice and wild-type mice between 30 and 42 days (wt/30 versus wt/42 and hm/30 versus hm/42). Some consistent changes in mRNA expression patterns were found in the *Egr-1* knockout mice compared to wild-type mice. Whether these genes are directly involved in the temporarily enhanced axial eye growth, or represent just epiphenomena, remains undefined and needs to be determined by further studies.

### Microarray

#### Gene expression changes over time (p30 versus p42)

It was not surprising that in wild-type mice only 54 genes showed differential mRNA expression over time (wt/30 versus wt/42, see Appendix 1), since the mouse retina is generally considered to be mature at the age of about p21 [36]. In the homozygous *Egr-1* knockout mice, 215 genes (roughly four times as many) were differently expressed (hm/30 versus hm/42, Appendix 2). Eight genes showed similar changes in mRNA expression in both the homozygous and the wild-type *Egr-1* knockout mice (shown in italics and underlined in Appendix 1 and Appendix 2). They likely encode for proteins that are involved in normal retinal function and are not related to the abnormal ocular growth in the knockout mice.

The average mean fold-changes in this study were 1.48±0.41, which is in line with findings from other microarray studies [16,19,37,38]. It has to be kept in mind that global gene expression measurements in a heterogeneous tissue like the retina are difficult to interpret since large changes in mRNA expression in a subset of cells might be obscured by changes in the opposite direction in a different, perhaps even more abundant population of cells. For the same reason, large changes in mRNA expression in a rare cell type might go unnoticed because they generate only a small fraction of the total mRNA. In the future, quantitative immunohistochemistry might help to detect changes in gene products which are localized to certain cells types.

### Differences in gene expression in Egr-1 knockout mice and their wild-type

Thirteen genes were differentially expressed in knockout mice, compared to wild-type, at both ages tested (Table 2 and Table 3: genes are shown in italics and underlined). Except for dystrophin (*DMD*), the regulation of those genes was in the same direction at both ages. These genes are therefore most probably target genes of Egr-1 and are not directly related to the developmental changes observed in these mice. Nevertheless, these thirteen genes are of interest because they were not previously described as possible targets for the Egr-1 protein. Still, whether the interaction with the Egr-1 protein is direct or indirect, has yet to be experimentally validated using other techniques like chromatin immunoprecipitation. The *Egr-1* knockout mice used in our study contain several in-frame stop codons in the *Egr-1* coding sequence, upstream of the zinc finger DNA binding domain. Parts of the mRNA sequence of *Egr-1* can therefore still be transcribed but the stop-codons lead to the functional elimination of the protein Egr1. The truncated *Egr-1* mRNAs can still bind to the microarray. Upregulation of *Egr-1* mRNA itself in the knockout mice can be explained by the fact that Egr-1 can suppress its own expression, as has been shown in tissue culture [39]. A negative feedback mechanism has already been described elsewhere and can be seen for *Nab2* (Ngfi-A-binding protein-2) [40]. This protein was massively downregulated in *Egr-1* knockout mice and is assumed to be a major regulator of Egr-1 function, since it is induced by the same stimuli that induce Egr-1. Dystrophin (*DMD*) mRNA expression was lower at p30 in the homozygous mice, compared to the wild-type mice, and higher at p42. An enhanced DMD content may therefore be correlated with enhanced axial eye growth. DMD is a plasma membrane-associated cytoskeletal protein of the spectrin superfamily and its absence or functional deficiency is the cause of several types of muscular dystrophies in humans. In some of these patients, retinal function is affected as well, as reflected in a reduced b-wave in the electroretinograms (ERG). DMD isoforms have been localized to Müller cells and photoreceptor terminals, so the abnormality in the ERGs is most likely due to a disturbance of neurotransmission between photoreceptors and ON-bipolar cells [41]. Interestingly, the lack of the dystrophin isoform Dp71 leads to impaired clustering of two Müller glia cell proteins in mice, namely the inwardly rectifying potassium channel Kir4.1 and the water pore aquaporin 4 (AQP4) [42]. Both Kir4.1 and AQP4 have already been implicated in the development of myopia and their role as a conduit for movement of retinal fluid into the vitreous was suggested [43,44].

Protocadherin beta 9 (*Pcdhb9*) was the most heavily regulated gene in this study (wt/30 versus hm/30: 14-fold downregulated and wt/42 versus hm/42: 17-fold downregulated in the homozygous *Egr-1* knockout mice). Protocadherins are calcium-dependent cell–cell adhesion molecules. Their specific functions are unknown, but they most likely play a critical role in the establishment and function of specific cell-cell neural connections [45]. The massive downregulation of this gene suggests a tight control, either directly by Egr-1, or indirectly by other Egr-1-regulated genes.

### Ingenuity pathway analysis

The list of genes that were differentially expressed in the *Egr-1* knockout mice between the age of 30 and 42 days (hm/30 versus hm/42) was analyzed using Ingenuity Pathway Analysis Software. Many ubiquitous signaling pathways in the retina seem to be involved. Therefore, it is difficult to define those genes that are responsible for the development of the relative myopia in the 42-day-old *Egr-1* knockout mice. As can be seen in Figures 1A-C, genes that showed altered mRNA expression in this study were not the key regulators in the functional networks proposed by the software. As already mentioned above, these pathway schemes are based on known interactions between molecules. They provide only suggestions for possible interactions. Involvement of the intermediate proteins and co-factors in these pathways is, therefore, possible but not proven. Furthermore, since the changes in mRNA expression levels of any gene chosen from network A and B could not be validated in all cases, it is difficult to speculate about the involvement of these networks as illustrated in Figure 1.

The only data available on visual function in *Egr-1* knockout mice are from optomotor experiments. They did not show any differences in contrast sensitivity or spatial resolution to the wild-type [20]. Interestingly, the lack of Egr-1 does not seem to affect other sensory systems in the mouse, like the auditory system. Auditory function was studied by evoked brainstem responses (ABR) and otoacoustic emissions (DPOAE), but no differences were found between wild-types and knockout mice (Dr. Lukas Rüttiger, Tuebingen Hearing Research Centre [THRC], Tuebingen, Germany, personal communication 2008). Therefore the function of Egr-1 in the regulation of axial eye growth seems to be quite specific despite the fact that common signaling pathways were affected.

### Real-time RT–PCR

#### Validation of genes

The mRNA expression levels of four out of the ten tested genes could be validated (Figure 2). To be able to compare the relative expression levels as determined by microarray analysis (MA) and real-time PCR (PCR) easily, Figure 2 shows the results of MA and PCR experiments separately for each gene. Non-validated genes are not represented in this figure. The fold-changes determined by PCR and MA of *Narf*, *Ogdh*, and *Selenbp1* were very similar whereas for *Pcdhb9*, the magnitude of changes was severely overestimated by MA. Considering this fact, the involvement of network A and B is more questionable, since none of the genes tested with real-time PCR could be validated. On the other hand, only three genes were tested in each network, and others which would have been validated upon testing might have been overlooked. Our primers were designed to bind to the same part of the sequence that was detected by the oligonucleotide probes on the Affymetrix chip (to avoid detection of different isoforms) and should have been appropriate to validate the microarray results. The applied false-discovery-rate of 5% thus does not seem to reflect the true errors that are unavoidable in this technique. These observations confirm that validation of the results of microarray analyses by other techniques (in this case semiquantitative real-time RT–PCR) should be mandatory.

### Genes whose transcription could be validated

The functions of the three genes which could be validated with real-time PCR are described in more detail below:

#### Narf

The expression of Nuclear prelamin A recognition factor was found to be lowest in the 42 days old knockout mice. Prenylation and methylation occurs at the C-terminal end of proteins and was initially believed to be important only for membrane attachment. However, another role for prenylation appears to be the mediation of protein–protein interactions [46]. The only nuclear proteins known to be prenylated in mammalian cells are prelamin A- and B-type lamins. Lamins are fibrous proteins providing structural function and transcriptional regulation in the cell nucleus, but the cellular role of both the prenylated prelamin A precursor and Narf, which is known to bind to the farnesylated prelamin A, is unknown and its role in the retina is unclear.

#### Ogdh

Oxoglutarate dehydrogenase mRNA expression was significantly enhanced in 42-day-old knockout mice. Ogdh is a mitochondrial enzyme complex, comprising of three different subunits (Ogdh, Dld, Dlst) that converts 2-oxoglutatate into succinyl-CoA and carbon dioxide in the Krebs cycle. Its reduction in patients with Alzheimer disease suggests an altered metabolism of the nervous tissue [47]. Unfortunately, no information is available yet for the role of this enzyme in the retina, but it could well be that the changes in mRNA expression of this gene reflects changes in the metabolic rate of retinal cells in the homozygous *Egr-1* knockout mice.

#### Selenbp1

Egr-1 knockout mice showed a high expression of Selenbp1 at the age of 30 days, with a subsequent decline. Selenium binding protein has been shown in several studies to be downregulated in cancer [48,49]. A protective effect of selenium in preventing macular degeneration has also been shown [50] and another selenium transporter (selenoprotein P) has been found to be upregulated in the retina of chicks that were treated with either positive or negative lenses [51]. Selenbp1 expression can be blocked by TGF-β in smooth muscle cells [52] and this protein (TGF-β) has already been implicated in myopia [53,54] and in the regulation of programmed cell death in the retina [55]. The role of selenium and its associated binding proteins in the retina of *Egr-1* knockout mice merits further investigation.

### Involvement of network C

Although the genes for which expression changes could be validated belong to this network, the involvement of the “key molecules” presented in Figure 1C remains speculative. No mRNA expression changes of e.g., *VEGF*, *cFos*, or *EGF* were found in this study although oligonucleotides representing mRNAs of *VEGF*, *cFos*, *EGF,* and retinoic acid receptors are present on the Affymetrix chip used in this study. Obviously, the lack of functional Egr-1 protein did not affect mRNA expression levels of these genes.

Nevertheless, some of those molecules have already been proposed to play a role in the regulation of eye growth, or were shown to change in the retina in response to various optical stimuli [4-6,13,16,56-62]. The fact that they seem to link several factors that were found to have changed mRNA expression levels in *Egr-1* knockout mice is in line with the idea that these molecules are involved in the regulation of eye growth.

### Outlook

This study provided a list of genes that appear associated with Egr-1 signaling and/or altered eye growth in *Egr-1* knockout mice. It is difficult to define specific roles to those genes in eye growth in the *Egr-1* knockout mice. Analysis of possibly involved networks show that the lack of Egr-1 affects common pathways in the retina. Furthermore, other important factors (like cFos, VEGF, NfkB) may play a role as well, perhaps partially taking over the role of Egr-1 in the retina of *Egr-1* knockout mice. We believe that the microarray analysis is a powerful tool to detect NEW candidates, rather than to look at the “usual suspects.” It is, however, difficult to use the microarray technology in heterogeneous tissue like retina. There are now advanced methods available to focus on certain cell types (e.g., laser capture microdissection of fluorescent-activated cell sorting). Nevertheless, some interesting genes were found in this study. In addition, several direct or indirect target genes of Egr-1 could be identified, including the most prominently regulated gene protocadherin-beta 9. The list of differentially expressed genes is accessible online in the Gene Expression Omnibus (GEO) Database and might be useful not only to researchers in the field of myopia.

## Appendix 1. List of differentially expressed genes in the wild-type mice between p30 and p42 days (wt/30 versus wt/42).

To access the data, click or select the words “Appendix 1.” This will initiate the download of a (pdf) archive that contains the file. Affymetrix ID, gene symbol, gene title, fold change (FC) and p-values of both the wild-type mice and the homozygous *Egr-1* knockout mice are shown. Genes were sorted after GO annotations. Genes that were significantly changed (with a FC > 1.5 and p-value < 0.05) in both the wild-type mice and the homozygous *Egr-1* knockout mice are shown in italics and are underlined.

## Appendix 2. List of genes that were differentially expressed in the homozygous *Egr-1* knockout mice between p30 and p42 (hm/30 versus hm/42).

To access the data, click or select the words “Appendix 2.” This will initiate the download of an Excel (.xls) file that contains the file.Affymetrix ID, gene symbol, gene title, fold change (FC), and p-values of both the homozygous *Egr-1* knockout mice and the wild-type mice are shown. Genes were sorted after GO annotations. Genes that were significantly changed (with a FC >1.5 and p-value <0.05) in both the homozygous wild-type mice and the *Egr-1* knockout mice are shown in italics and are underlined. Genes that were chosen for real-time RT–PCR validation are shown in bold.
